# Study of the Risk Factors of Ectopic Pregnancy and Various Modalities in Treatments in a Tertiary Care Centre: A Retrospective Study

**DOI:** 10.7759/cureus.113845

**Published:** 2026-08-03

**Authors:** Ravindra S Pukale, Spoorthi V Raj, Indumathi M

**Affiliations:** 1 Obstetrics and Gynaecology, Adichunchanagiri Institute of Medical Sciences, Adichunchanagiri University, Mandya, IND; 2 Obstetrics and Gynaecology, BGS Medical College and Hospital, Adichunchanagiri University, Bengaluru, IND

**Keywords:** ectopic pregnancy, laparotomy, methotrexate, risk factors, tubal pregnancy

## Abstract

Introduction: Ectopic pregnancy is a significant cause of first-trimester morbidity and mortality. Despite advances in diagnostic modalities, delayed presentation remains common in many settings, leading to increased complications and the need for surgical intervention. The present study aimed to evaluate the risk factors, clinical profile, diagnostic findings, and various modalities of management of ectopic pregnancy in a tertiary care centre.

Materials and methods: A retrospective observational study was conducted over a period of three years from May 2022 to May 2025 at Adichunchanagiri Institute of Medical Sciences, Adichunchanagiri University, Karnataka, India. A total of 45 cases of ectopic pregnancy were included. Data regarding demographic profile, risk factors, clinical presentation, ultrasonographic findings, management, intraoperative details, and outcomes were collected from hospital records and analysed using IBM SPSS Statistics version 26.0. Results were expressed in N (%) format.

Results: The incidence of ectopic pregnancy was 45/2398 (1.87%). The majority of patients were in the age group of 26-30 years (20; 44.4%) and were multigravida (27; 60.0%). Common risk factors included previous lower-segment caesarean section (LSCS) (18; 40.0%) and abortions (16; 35.6%), while 13 (28.9%) had no identifiable risk factors. Abdominal pain was the most common symptom (38; 84.4%). Tubal ectopic pregnancy predominated in 37 (82.2%), with ampullary region involvement in 20 (54.1%). Ruptured ectopic pregnancy was observed in 35 (77.8%) cases. Surgical management was required in 41 (91.1%) patients, with laparotomy in 25 (61.0%) cases. Blood transfusion was required in 30 (66.7%) patients. There was no mortality.

Conclusion: Ectopic pregnancy continues to present as a surgical emergency due to delayed diagnosis. Early detection and timely intervention can reduce morbidity and allow for conservative management in selected cases.

## Introduction

Ectopic pregnancy is defined as implantation of a fertilized ovum outside the endometrial cavity, most commonly within the fallopian tube [1]. It represents a significant cause of maternal morbidity and mortality, particularly in the first trimester of pregnancy [2]. Despite advances in diagnostic techniques and management strategies, ectopic pregnancy continues to pose a clinical challenge due to its varied presentation and potential for life-threatening complications, such as tubal rupture and haemorrhagic shock [3].

The incidence of ectopic pregnancy has shown a rising trend globally, which may be attributed to increased prevalence of risk factors such as pelvic inflammatory disease, prior tubal surgeries, assisted reproductive techniques, and higher rates of caesarean sections [4]. However, a substantial proportion of cases occur in women without documented or readily identifiable risk factors, making early diagnosis difficult. The classical triad of amenorrhoea, abdominal pain, and vaginal bleeding is not consistently present, further complicating timely recognition [5].

With the advent of transvaginal ultrasonography and sensitive serum β-hCG assays, early diagnosis of ectopic pregnancy has improved significantly, allowing for conservative management in selected cases [6-8]. Medical management using methotrexate and expectant management are now viable options in haemodynamically stable patients with early unruptured ectopic pregnancy [9]. Nevertheless, in many developing settings, delayed presentation remains common, leading to a higher proportion of ruptured ectopic pregnancies requiring emergency surgical intervention [10,11].

Understanding the demographic profile, associated risk factors, clinical presentation, diagnostic findings, and management outcomes is essential for improving early detection and reducing morbidity. This study aimed to evaluate the risk factors, clinical profile, diagnostic findings, and various modalities of management of ectopic pregnancy in a tertiary care centre.

## Materials and methods

This retrospective observational study was conducted at Adichunchanagiri Institute of Medical Sciences, Adichunchanagiri University, Karnataka, India, over a period of three years, from May 2022 to May 2025. As this was a retrospective record-based study, no formal sample size calculation was performed. All consecutive patients diagnosed with ectopic pregnancy during the study period who fulfilled the eligibility criteria were included, resulting in a final study sample of 45 cases. Institutional Ethics Committee approval from the Institutional Ethics Committee of Adichunchanagiri Institute of Medical Sciences was obtained prior to commencement of the study (IEC No: AIMS/IEC/227/2025), and confidentiality of patient data was strictly maintained throughout the study.

All patients with a confirmed diagnosis of ectopic pregnancy based on clinical findings, ultrasonography, and/or intraoperative confirmation were included. Patients managed by surgical, medical, or expectant modalities were considered. Cases with incomplete clinical details or inconclusive diagnosis were excluded to maintain data integrity. Diagnosis was primarily based on a combination of clinical presentation, transabdominal or transvaginal ultrasonography findings, and serum β-hCG levels wherever available.

Data were collected from hospital medical records, operative notes, and discharge summaries using a predesigned structured proforma. Variables recorded included demographic characteristics such as age, gravidity, and interval since last childbirth; obstetric and gynaecological risk factors documented in the medical records, including previous caesarean section, history of abortions, tubal surgeries, IUCD usage, and prior ectopic pregnancy. Clinical presentation at admission, including symptoms, signs, and haemodynamic status, was documented. Ultrasonographic findings such as presence of adnexal mass, free fluid, and gestational sac were noted. In surgically managed patients, intraoperative findings including site of ectopic pregnancy, laterality, rupture status, and estimated blood loss were recorded.

Management modalities were categorized into surgical, medical, and expectant approaches. Details of surgical intervention, including the surgical approach (laparoscopy or laparotomy) and the operative procedure performed (such as salpingectomy, salpingostomy, salpingo-oophorectomy, cornual resection, or other procedures where applicable), were documented. Medical management protocols, including administration of methotrexate and follow-up with serial β-hCG levels, were also noted. Outcomes assessed included requirement of blood transfusion, postoperative complications, need for intensive care unit admission, and mortality.

The collected data were entered into Microsoft Excel (Microsoft Corp., USA) and subsequently analysed using IBM SPSS Statistics version 26.0 (IBM Corp., Armonk, NY, USA). Descriptive statistical analysis was performed, and categorical variables were expressed as numbers and percentages. Continuous variables were summarized using mean and standard deviation where appropriate.

## Results

A total of 2,398 deliveries were recorded during the study period, among which 45 cases were diagnosed as ectopic pregnancies, yielding an incidence of 45/2398 (1.87%) (Table 1).

**Table 1:** Incidence of ectopic pregnancy Data are presented as number or incidence (%). Incidence was calculated as the number of ectopic pregnancies divided by the total number of deliveries during the study period.

Parameter	Value
Total deliveries	2,398
Total ectopic pregnancies	45
Incidence	45/2,398 (1.87%)

The majority of patients were in the age group of 26-30 years (20; 44.4%), followed by 31-35 years (9; 20.0%) and 21-25 years (8; 17.8%). Extremes of age were less commonly affected, with ≤20 years accounting for three (6.7%) and >35 years for five (11.1%). Most patients were multigravida, with G3 constituting 14 (31.1%) and G2 constituting 13 (28.9%), while primigravida accounted for eight (17.8%). Regarding interval since the last childbirth, the most common duration was one to two years (15; 33.3%), followed by >10 years (6; 13.3%) and three to four years (6; 13.3%) (Table 2).

**Table 2:** Demographic and obstetric characteristics (n = 45) Data are presented as n (%). Abbreviations: G, gravidity.

Variable	Category	N (%)
Age (years)	≤20	3 (6.7%)
	21–25	8 (17.8%)
	26–30	20 (44.4%)
	31–35	9 (20.0%)
	>35	5 (11.1%)
Gravidity	Primigravida	8 (17.8%)
	G2	13 (28.9%)
	G3	14 (31.1%)
	G4	7 (15.6%)
	≥G5	3 (6.7%)
Interval since the last childbirth	<1 year	3 (6.7%)
	1–2 years	15 (33.3%)
	3–4 years	6 (13.3%)
	5–6 years	2 (4.4%)
	7–8 years	2 (4.4%)
	9–10 years	3 (6.7%)
	>10 years	6 (13.3%)

The majority of patients presented between 6-7+6 weeks of gestation (19; 42.2%), followed by 8-9+6 weeks (15; 33.3%) and 4-5+6 weeks (10; 22.2%). Only one (2.2%) patient presented beyond 10 weeks, while none presented before four weeks (Table 3).

**Table 3:** Gestational age at presentation (n = 45) Data are presented as n (%). Gestational age is expressed in completed weeks of pregnancy.

Gestational age	N (%)
≤4 weeks	0 (0%)
4–5+6 weeks	10 (22.2%)
6–7+6 weeks	19 (42.2%)
8–9+6 weeks	15 (33.3%)
≥10 weeks	1 (2.2%)
Total	45 (100%)

Among the risk factors, previous lower-segment caesarean section was the most common, seen in 18 (40.0%) patients, followed by history of abortions in 16 (35.6%) and prior tubal surgery in nine (20.0%). Other risk factors, such as intrauterine contraceptive device (IUCD) use, previous ectopic pregnancy, and recanalization, were each observed in one (2.2%) case. Among the available medical records, no documented risk factors were identified in 13 (28.9%) patients (Table 4).

**Table 4:** Risk factors for ectopic pregnancy (n = 45) Data are presented as n (%). Patients may have had more than one risk factor; therefore, percentages may not total 100%. Abbreviations: LSCS, lower-segment caesarean section; IUCD, intrauterine contraceptive device

Risk factor	N (%)
Previous LSCS	18 (40.0%)
Abortions	16 (35.6%)
Tubal surgery	9 (20.0%)
IUCD	1 (2.2%)
Previous ectopic pregnancy	1 (2.2%)
Recanalization	1 (2.2%)
No documented risk factors	13 (28.9%)

Abdominal pain was the most common presenting symptom, observed in 38 (84.4%) patients, followed by bleeding per vagina in 17 (37.8%) and amenorrhoea in 12 (26.7%). The classical triad was present in only three (6.7%) cases. Haemorrhagic shock was noted in two (4.4%) patients. Other associated symptoms included vomiting in eight (17.8%), loss of consciousness in two (4.4%), weakness in two (4.4%), breathlessness in one (2.2%), and shoulder tip pain in one (2.2%). Incidental diagnosis was made in four (8.9%) cases (Table 5).

**Table 5:** Clinical presentation (n = 45) Data are presented as n (%). Patients may have presented with multiple clinical features; therefore, percentages may not total 100%.

Clinical feature	N (%)
Abdominal pain	38 (84.4%)
Bleeding per vagina	17 (37.8%)
Amenorrhoea	12 (26.7%)
Classical triad	3 (6.7%)
Haemorrhagic shock	2 (4.4%)
Vomiting	8 (17.8%)
Loss of consciousness	2 (4.4%)
Breathlessness	1 (2.2%)
Weakness/fatiguability	2 (4.4%)
Shoulder tip pain	1 (2.2%)
Incidental diagnosis	4 (8.9%)

Tubal ectopic pregnancy was the predominant type, accounting for 37 (82.2%) cases, followed by ovarian ectopic in two (4.4%). Other rare types, including cervical, pregnancy of unknown location, scar ectopic, heterotopic, and tubo-ovarian pregnancies, were each observed in one (2.2%) case. Among tubal ectopic pregnancies, the ampullary region was the most commonly involved site (20; 54.1%), followed by the isthmo-ampullary junction (7; 18.9%). Right-sided involvement was more common (23; 62.2%), compared to the left (14; 37.8%). A majority of cases presented as ruptured ectopic pregnancies (35; 77.8%), while 10 (22.2%) were unruptured (Table 6).

**Table 6:** Type, site, and status of ectopic pregnancy (n = 45) Data are presented as n (%). Percentages for tubal site and laterality are calculated using the number of tubal ectopic pregnancies (n = 37) as the denominator.

Variable	Category	N (%)
Type	Tubal	37 (82.2%)
	Ovarian	2 (4.4%)
	Cervical	1 (2.2%)
	Pregnancy of unknown location	1 (2.2%)
	Scar ectopic	1 (2.2%)
	Heterotopic	1 (2.2%)
	Tubo-ovarian	1 (2.2%)
Tubal site (n = 37)	Ampullary	20 (54.1%)
	Isthmo-ampullary	7 (18.9%)
	Isthmus	3 (8.1%)
	Cornual	3 (8.1%)
	Others	4 (10.8%)
Laterality (tubal, n = 37)	Right	23 (62.2%)
	Left	14 (37.8%)
Status (n = 45)	Ruptured	35 (77.8%)
	Unruptured	10 (22.2%)

On ultrasonography, free fluid in the pelvis was the most common finding, seen in 31 (68.9%) patients, followed by adnexal mass in 21 (46.7%) and gestational sac in adnexa in 13 (28.9%). Cardiac activity was noted in five (11.1%) cases. Other findings, such as heterotopic pregnancy, suspected haemorrhagic cyst, tubo-ovarian mass, Mullerian anomaly, thickened endometrium, scar ectopic, and non-definitive location, were each seen in one (2.2%) case, while uterine fibroids were noted in two (4.4%) patients (Table 7).

**Table 7:** Ultrasound findings (n = 45) Data are presented as n (%). Patients may have had more than one ultrasonographic finding; therefore, percentages may not total 100%.

Finding	N (%)
Free fluid in pelvis	31 (68.9%)
Adnexal mass	21 (46.7%)
Gestational sac in adnexa	13 (28.9%)
Cardiac activity	5 (11.1%)
Heterotopic pregnancy	1 (2.2%)
Suspected haemorrhagic cyst	1 (2.2%)
Suspected tubo-ovarian mass	1 (2.2%)
Uterine fibroids	2 (4.4%)
Mullerian anomaly	1 (2.2%)
Thickened endometrium	1 (2.2%)
Scar ectopic	1 (2.2%)
Location not definitive	1 (2.2%)
Normal scan	1 (2.2%)

Among surgically managed patients, laparotomy was performed in 25 (61.0%) cases and laparoscopy in 16 (39.0%). The most commonly performed operative procedure was salpingectomy, while other procedures were undertaken according to the intraoperative findings (Table 8).

**Table 8:** Management and surgical details Data are presented as n (%). Percentages for surgical approach are calculated among surgically managed patients (n = 41).

Variable	Category	N (%)
Management (n = 45)	Surgical	41 (91.1%)
	Medical	3 (6.7%)
	Expectant	1 (2.2%)
Surgical approach (n = 41)	Laparoscopy	16 (39.0%)
	Laparotomy	25 (61.0%)

Among the surgically managed patients (n = 41), intraoperative blood loss was most commonly in the range of 100-500 mL (14; 34.1%), followed by <100 mL in eight (19.5%) and 500-1000 mL in six (14.6%). Higher blood loss of 1500-2000 mL and >2000 mL was observed in five (12.2%) cases each (Table 9).

**Table 9:** Intraoperative blood loss (n = 41) Data are presented as n (%). Blood loss was assessed only in patients who underwent surgical management.

Blood loss	N (%)
<100 mL	8 (19.5%)
100–500 mL	14 (34.1%)
500–1000 mL	6 (14.6%)
1000–1500 mL	3 (7.3%)
1500–2000 mL	5 (12.2%)
>2000 mL	5 (12.2%)
Total	41 (100%)

Blood transfusion was required in 30 (66.7%) patients, while 15 (33.3%) did not require transfusion. Among those transfused, the most common requirement was one unit of packed red blood cells (PRBC) in 14 (31.1%) patients, followed by two units in eight (17.8%) and three units in six (13.3%) cases. ICU admission was required in three (6.7%) patients. Postoperative complications included fever in two (4.4%) and pulmonary complications in two (4.4%) patients. No mortality was observed in the study (Table 10).

**Table 10:** Blood transfusion and outcomes (n = 45) Data are presented as n (%). Postoperative outcomes are not mutually exclusive; therefore, percentages may not total 100%. Abbreviations: PRBC, packed red blood cells; ICU, intensive care unit

Variable	Category	N (%)
Transfusion required	Yes	30 (66.7%)
	No	15 (33.3%)
PRBC units	1 unit	14 (31.1%)
	2 units	8 (17.8%)
	3 units	6 (13.3%)
	4 units	2 (4.4%)
Outcomes	ICU admission	3 (6.7%)
	Fever	2 (4.4%)
	Pulmonary complications	2 (4.4%)
	Mortality	0 (0%)

## Discussion

The incidence of ectopic pregnancy in the present study was 45/2398 (1.87%), which is comparable to other tertiary care studies. A recent Indian study by Sharma S et al. reported 68/3628 (1.87%) cases, showing an identical incidence, while another study by Sharma A et al. reported an incidence of approximately 1.45% [12,13].

In the present study, the majority of patients were in the age group of 26-30 years (20; 44.4%), which is consistent with Sharma S et al., where 46/68 (67.64%) patients were in the 20-30 years' group [12]. Similarly, Wakode et al. reported 129 cases accounting for 65.48% in the 21-30 years' age group, indicating that ectopic pregnancy predominantly affects women in peak reproductive age [14]. Multigravida predominance in the present study (G2+G3: 27/45; 60.0%) is comparable to Sharma S et al., where multiparous women constituted 50/68 (73.53%) [12].

Regarding risk factors, previous LSCS 18/45 (40.0%), abortions 16/45 (35.6%), and tubal surgery 9/45 (20.0%) were predominant in the present study. Similar trends have been reported in other studies where prior uterine procedures and tubal damage significantly contribute to ectopic pregnancy. In a study of 110 cases, 25/110 (22.73%) patients had no documented risk factors, which is comparable to the present study where 13/45 (28.9%) had no documented risk factors based on the available medical records [15]. Wakode et al. also reported absence of documented risk factors in 67 (34.01%) cases, further supporting that a substantial proportion of ectopic pregnancies occur without documented or readily identifiable risk factors [14].

Abdominal pain was the most common presenting symptom in the present study, 38/45 (84.4%), followed by bleeding per vagina 17/45 (37.8%) and amenorrhoea 12/45 (26.7%). Comparable findings were reported by Sharma S et al., where abdominal pain was seen in 55/68 (80.88%) and amenorrhoea in 65/68 (95.58%) cases [12]. Haemodynamic instability was noted in 2/45 (4.4%), comparable to 5/68 (7.35%) reported by Sharma S et al. [12].

Tubal ectopic pregnancy was the most common type in the present study, 37/45 (82.2%), which is consistent with Sharma S et al. reporting tubal ectopic pregnancy in 65/68 (95.58%) cases [12]. Among tubal pregnancies, ampullary involvement was most frequent, 20/37 (54.1%), similar to Sharma S et al. reporting 38/68 (55.88%) and Wakode et al. reporting 126 (63.95%) cases in the ampullary region [12,14]. Right-sided predominance was observed in 23/37 (62.2%) cases compared to left-sided involvement in 14/37 (37.8%), which is consistent with previous literature. A higher proportion of ruptured ectopic pregnancies was observed in the present study, 35/45 (77.8%), compared to Wakode et al. reporting rupture in 117/197 (59.39%) cases, suggesting relatively delayed presentation in the present setting [14].

Ultrasonography findings in the present study showed free fluid in 31/45 (68.9%) and an adnexal mass in 21/45 (46.7%). Similar findings were observed in other studies where ultrasonography plays a crucial role in early diagnosis and identification of haemoperitoneum [15]. Detection of an adnexal gestational sac in 13/45 (28.9%) cases in the present study is comparable with published literature, where visualization of a definite extrauterine gestational sac is observed only in a minority of ectopic pregnancies, with most cases presenting as nonspecific adnexal masses on ultrasonography [16].

Surgical management was the predominant modality in the present study, 41/45 (91.1%), similar to other studies where surgery remains the mainstay due to late presentation. Wakode et al. reported surgical management in 112/197 (56.79%) cases, with salpingectomy being the most common procedure [14]. Higher surgical rates in the present study reflect the higher proportion of ruptured ectopic pregnancies.

Intraoperative blood loss in the present study was most commonly 100-500 mL in 14/41 (34.1%) cases, with significant blood loss (>1000 mL) in 13/41 (31.7%) patients. Blood transfusion was required in 30/45 (66.7%) cases, which is higher compared to Sharma S et al. reporting transfusion in 37/68 (54.41%) patients [12]. ICU admission was required in 3/45 (6.7%) patients, comparable to 4/68 (5.8%) reported by Sharma S et al. [12]. No mortality was observed in the present study, consistent with recent literature demonstrating improved outcomes with timely intervention [12].

Strengths and limitations

The present study provides a comprehensive evaluation of ectopic pregnancy in a tertiary care setting, encompassing detailed analysis of demographic characteristics, risk factors, clinical presentation, diagnostic findings, management modalities, intraoperative details, and outcomes, thereby offering a holistic clinical perspective. Inclusion of all diagnosed cases over a defined period and standardized data extraction enhances the internal consistency of the study. However, the retrospective design limits control over data completeness and may introduce information bias. The relatively small sample size and single-centre setting restrict the generalizability of the findings. In addition, the lack of long-term follow-up precludes assessment of future fertility outcomes and recurrence risk.

## Conclusions

Ectopic pregnancy remains a significant obstetric emergency associated with substantial maternal morbidity, largely due to delayed presentation. The present study demonstrates that most patients were women of reproductive age, with previous caesarean section and history of abortions being the most commonly documented risk factors, although a considerable proportion had no documented risk factors in the available medical records. Abdominal pain was the predominant presenting symptom, and the high frequency of ruptured ectopic pregnancies resulted in surgical management in the majority of cases. These findings underscore the importance of maintaining a high index of clinical suspicion and promoting early diagnosis through timely ultrasonography and serum β-hCG assessment, which may facilitate conservative management in appropriate patients and reduce associated morbidity.
